# The Brain Health Champion Study: A Health Coaching Intervention with Mobile Technology in Older Adults with Mild Cognitive Impairment or Risk Factors for Dementia

**DOI:** 10.1002/alz70860_106798

**Published:** 2025-12-23

**Authors:** Kayla Riera, Kirk R Daffner, Brittany M McFeeley, Seth A Gale, George Ghorayeb

**Affiliations:** ^1^ Brigham and Women's Hospital, Boston, MA, USA

## Abstract

**Background:**

Cumulative evidence suggests that adhering to brain‐healthy behaviors can decrease risk of cognitive decline and dementia. We previously demonstrated that a health coaching intervention, including weekly phone calls, facilitated adherence to lifestyle recommendations in older adults with mild cognitive impairment (MCI or mild dementia. The current study extends this research to include cognitively normal, older adults at risk for dementia.

**Method:**

Participants, age 60‐79, with mild cognitive impairment (MCI) due to suspected Alzheimer, cerebrovascular, or “mixed” disease, or older adults with dementia risk factors were randomized to Brain Health Champion (BHC) intervention or a counseling and education (CE) control. In BHC, participants set personalized goals, supported by a health coach and reinforced by weekly video calls and a dietitian consultation. In CE, educational materials were provided every six weeks to supplement usual care. Changes in diet, subjective cognitive concerns, physical activity, social/cognitive engagement, neurocognitive test scores, and behavioral health metrics were measured over six months using questionnaires, wearable fitness trackers, and photographed food logs.

**Result:**

55 participants (17 MCI, 38 at risk) enrolled in the study, with 45 completers (23 BHC, 22 CE). BHC participants reported significantly increased adherence to a Mediterranean diet and reduction of subjective cognitive concerns. Within the BHC group, there were trends toward intervention‐related improvement in physical activity, mood, cognitive activity, and quality of life, that were not observed in the CE group. Participant feedback pointed toward increased brain health knowledge, satisfaction with the coach relationship, and positive overall change in behavior in the BHC group. CE participants did not report comparable satisfaction with the educational materials nor as much readiness to commit to lifestyle changes.

**Conclusion:**

A 6‐month, mobile technology‐enhanced coaching program (BHC) appears effective in promoting brain‐healthy behaviors and is feasible for at‐risk older adults or those with MCI. Participation in this clinic‐embedded brain health‐promoting program tended to augment physical and cognitive activity, mood, quality of life, and adherence to a Mediterranean diet, which are lifestyle changes associated with decreased risk of cognitive decline. In contrast, supplementing usual provider care with written educational materials did not lead to meaningful behavioral changes.

